# Core Evidence‐Based Practice Competencies and Learning Outcomes for European Nurses: Consensus Statements

**DOI:** 10.1111/wvn.12506

**Published:** 2021-05-24

**Authors:** Jakub Dolezel, Renata Zelenikova, Stefano Finotto, Daniela Mecugni, Athina Patelarou, Mariusz Panczyk, Maria Ruzafa‐Martínez, Antonio Jesús Ramos‐Morcillo, Brigita Skela‐Savič, Joanna Gotlib, Evridiki Patelarou, Marta Smodiš, Darja Jarosova

**Affiliations:** ^1^ Department of Nursing and Midwifery Faculty of Medicine University of Ostrava Ostrava Czech Republic; ^2^ University of Modena e Reggio Emilia Modena Italy; ^3^ Department of Nursing Faculty of Health Sciences Hellenic Mediterranean University Crete Greece; ^4^ Department of Education and Research in Health Sciences Faculty of Health Science Medical University of Warsaw Warsaw Poland; ^5^ Faculty of Nursing University of Murcia Murcia Spain; ^6^ Angela Boškin Faculty of Health Care Jesenice Slovenia

**Keywords:** evidence‐based practice, competency, consensus, Delphi survey, learning outcome, learning, nursing education, nurse

## Abstract

**Background:**

Consensus on evidence‐based practice (EBP) competencies and associated learning outcomes for registered nurses has not yet been achieved in the European context.

**Aims:**

To establish a set of core EBP competencies for nurses and the most important EBP learning outcomes encompassing attitudes, knowledge, and skills dimensions for implementation into nursing education in European countries.

**Methods:**

A multi‐phase modified Delphi survey was conducted: Phase 1, a literature review; Phase 2, a two‐round consensus of experts; and Phase 3, a Delphi survey. Experts from six European countries participated.

**Results:**

In Phase 1, 88 records were selected and 835 statements extracted, which were grouped according to the seven steps of EBP. After removing 157 duplicates, the remaining competencies (*n* = 678) were evaluated in Phase 2. Then, a two‐round expert consensus was reached, with 24 competencies and 120 learning outcomes identified and divided into affective, cognitive, and skills domains. In Phase 3, based on a Delphi survey expert consensus, all evaluated statements were included in a final set of competencies and learning outcomes. Only two learning outcomes were recommended for allocation to a different domain, and four were reformulated as suggested, with no further changes to the others.

**Linking Evidence to Action:**

The set of EBP competencies and learning outcomes can guide nurse educators, managers, and EBP stakeholders in the development of content that incorporates EBP knowledge, skills, and attitudes into educational programs. Prioritizing the EBP competencies and learning outcomes that are most necessary and adapting them to every context will provide healthcare organizations with guidelines for enhancing the continuing education of nurses. These results could facilitate the development of effective tools for assessing nursing students’ and nurses’ perception of competencies required for EBP processes.

## BACKGROUND

A thorough integration of the best scientific evidence into daily practice is key to effective improvements in quality of care and patient outcomes (Saunders, Gallagher‐Ford, & Vehviläinen‐Julkunen, 2019). The World Health Organization (World Health Organization, 2017), therefore, states that it is imperative for countries in the WHO European region to consider the benefits of evidence‐based practice (EBP) and to focus on continuous improvements in quality of care. As the largest group of healthcare professionals, nurses play a key role in providing effective, safe, and evidence‐based care, which requires the translation of research results into EBP (World Health Organization, 2017). To ensure safe and quality nursing practice, the acquisition of EBP competency is essential (Orta et al., 2016); however, most nurses are not prepared for EBP (Oh et al., 2010; Patelarou et al., 2017; Saunders & Vehviläinen‐Julkunen, 2016). The lack of preparation is demonstrated through insufficient knowledge, values, and competencies for understanding and using EBP (Skela‐Savič, Hvalič‐Touzery, & Pesjak, 2017). Although nurses are familiar with the concept of EBP, have positive attitudes toward it, and believe that it may improve the quality of care and patient outcomes, they still perceive their own EBP skills to be inadequate and do not feel qualified to apply EBP in their work (Melnyk et al., 2018; Skela‐Savič et al., 2016; Zeleníková et al., 2016).

Expectations of improvement in EBP quality generate new professional competencies that allow healthcare professionals and organizations to clarify performance expectations regarding EBP and succinctly outline the expected competencies for successful application of best evidence to daily care (Saunders et al., 2019). As the education of nurses has developed from hospital‐based training to a competence‐based curriculum, set core competencies have become crucial for effective nursing training (Reeves, Fox, & Hodges, 2009). The 2015 Competency Framework, based on the European directive 2013/55/EU, recognized implementation of scientific findings into EBP as a central competency of the undergraduate education of nurses in the European Union (European Federation of Nurses Associations, 2015). In addition, the European Federation of Nurses Associations (2014) defined four categories of nursing care providers in a document entitled “EFN Matrix on the Four Categories of the Nursing Care Continuum,” which also introduced EBP tasks at the level of specialist nurse and advanced practice nurse. EBP has also been recognized as a central competency by the Quality and Safety Education for Nurses (QSEN) project (QSEN, 2020), a global nursing initiative whose purpose is to meet the challenge of preparing future nurses with the knowledge, skills, and attitudes necessary to continuously improve the quality and safety of the healthcare system they work in. Together with representatives from 11 other professional organizations, the QSEN Institute has defined quality and safety competencies for nursing and has proposed targets for the knowledge, skills, and attitudes to be developed in nursing graduate programs for each competency. In addition to competencies in patient‐centered care, teamwork and collaboration, quality improvement, safety, and informatics, they also included competencies for EBP (Cronenwett et al., 2007; QSEN, 2020).

### Conceptual Framework

There is no single definition for the concept of competency. For the purpose of this study, we considered competency as “the capacity of nurses to integrate cognitive, affective, and psychomotor abilities in nursing care provision” (Miller, Hoggan, Pringle, & West, 1988). Similarly, Leung, Trevena, and Waters (2016) view competency as the combination of complex attributes of knowledge, skills, and attitudes with the ability to make professional judgments and to perform intelligently in specific situations. In the field of education, the relationship between competencies and learning outcomes has been widely discussed. It has been suggested that competencies with a narrow focus could be regarded as learning outcomes (Kennedy, Hyland, & Ryan, 2009). However, others posit that learning outcomes support the competencies, are of greater detail, and form the basis of both learning and assessment (Oliver et al., 2008). Therefore, to ensure clarity of meaning, it has been recommended to translate competencies into learning outcomes—that is, express the required competency in terms of the students’ or professionals’ attainment of specific learning outcomes (Kennedy et al., 2009).

Generally, EBP competencies arise from key EBP principles and steps that are universal (Saunders et al., 2019). Laibhen‐Parkes (2014) suggests that EBP competency is the ability to ask clinically relevant questions for the purposes of acquiring, appraising, applying, and assessing multiple sources of knowledge within the context of caring for a particular patient, group, or community. Over the last decade, several national EBP competency frameworks have emerged, with the United States as a leading pioneer. Stevens (2009) reported a national consensus on essential competencies for EBP in nursing, reached through a process involving a panel of experts. These original competency statements were classified across the undergraduate, master, and doctoral levels of nursing preparation and organized into five stages of knowledge transformation, according to the ACE Star Model of Knowledge Transformation (Stevens, 2004). More recently, Melnyk, Gallagher‐Ford, Long, and Fineout‐Overholt (2014) developed a new set of EBP competencies for practicing registered nurses and advanced practice nurses in real‐world healthcare settings. These competencies are more focused on developing practice capabilities that nurses need for implementing EBP. The practice‐focused EBP competencies served as a starting point for establishing key content consensus on the essential EBP competencies for registered and advanced practice nurses among Finnish nurse panelists (Saunders et al., 2019). A study by Australian authors Leung et al. (2016) designed a competency framework for measuring EBP knowledge and skills based on the five‐step EBP model. Another set of core EBP competencies for healthcare professionals was identified using a Delphi survey, following a systematic literature review (Albarqouni, Hoffmann, Straus, et al., 2018).

Although previous work can be a useful starting point, in European health care, EBP is rarely an integral part of patient care or nursing education curricula (Ruzafa‐Martínez, 2019). Therefore, the main and most urgent task of working effectively to develop and provide professional education that facilitates the implementation of EBP still remains (Lehane et al., 2019). Agreement on EBP competencies and associated learning outcomes for registered nurses has not yet been achieved in the European context. Stakeholders in academic and clinical settings need a properly constructed set of competencies and learning outcomes outlining what nursing students and nurses should know, understand, and ﻿be able to do (and to what degree of proficiency), using language and contexts that indicate the level at which they will be assessed. The present proposal focuses on providing these outputs through the collaboration of six European higher education institutions participating in a project funded by the European Erasmus+ Programme (Ruzafa‐Martínez, 2019).

### Aim

The aim was to establish a set of core EBP competencies for nurses and the most important EBP learning outcomes encompassing attitudes, knowledge, and skills dimensions for implementation into nursing education in European countries, including undergraduate, graduate, and continuing education programs.

## METHODS

### Design

A multi‐phase modified Delphi survey was implemented via three phases: Phase 1 was literature review on EBP competencies; Phase 2 consisted of two rounds of expert consensus for prioritizing the most essential EBP competencies and learning outcomes; and Phase 3 instituted a Delphi survey to establish a final set of core EBP competencies and learning outcomes for nurses.

### Phase 1: A Literature Review on EBP Competencies

In Phase 1 (January–February 2019), a literature review was conducted to identify and analyze studies focusing on EBP competencies for nurses. Research teams from the Czech Republic, Greece, Italy, Poland, Slovenia, and Spain participated in the review. Studies were identified using a search strategy and predefined criteria in the bibliographic databases CINAHL Plus with Full Text, SpringerLink, Cochrane Library, ProQuest, ScienceDirect, Web of Science, SCOPUS, EBSCO, PubMed, Embase, and PsycINFO, as well as in the national databases of all participating countries. Each database was searched by two researchers from the different countries. For the present study, a Data Extraction Protocol was created (Table S1).

Using content analysis (Bengtsson, 2016), two researchers (JD and DJ) identified EBP competencies for nurses in the studies and grouped them according to the seven steps of EBP (Melnyk & Fineout‐Overholt, 2015). Subsequently, the set of competencies was revised (MRM and AJRM), and duplicate and irrelevant items were excluded.

### Phase 2: Two‐Round Expert Consensus to Prioritize the Most Essential EBP Competencies and Learning Outcomes

In the first round (April 2019), the project research team, consisting of two researchers from each participating country (12 researchers total), analyzed and assessed EBP competency statements, rating the adequacy of all items to their EBP steps, from 0 points (completely inadequate) to 100 points (completely adequate). They also evaluated the clarity and intelligibility of the wording for the competency statements. An EBP statement was maintained in the selected domain when a predefined consensus level of at least 70% of the experts was achieved. The assessed statements were revised, and some of the items were reworded and reassigned to other EBP steps, as necessary.

In the second round (May–June 2019), the research team reviewed the selected statements and allocated them to the corresponding affective, cognitive, skills, or practical domains. If necessary, statements were rewritten or new ones were added. According to the conceptual framework adopted in the study, competencies were considered to be statements of a general nature consisting of a subset of learning outcomes, as defined by Kennedy et al. (2009). A qualitative evaluation of the results allowed identification of two levels of specificity in the statements selected. Some were written as practical competencies or behavioral actions coinciding with statements allocated to the practical domain, whereas the more specific statements were assigned to the other domains (affective, cognitive, and skills) and deemed learning outcomes. Consequently, the research team agreed to consider statements from the practical domain (24 items in total) as specific competencies. The remaining statements were deemed learning outcomes, which were assessed in the next phase.

### Phase 3: A Delphi Survey to Establish a Final Set of Core EBP Competencies and Learning Outcomes for Nurses

The set of core EBP competencies for nurses, together with the learning outcomes selected during Phase 2, were assessed using a Delphi survey (McPherson, Reese, & Wendler, 2018) by a selection of experts from all the participating countries. Experts for the survey panel were designated in accordance with predefined criteria: healthcare professionals nationally renowned for EBP or the development of instruments, from different practice profiles and geographical areas in Europe. Members of the research team did not take part in the Delphi survey. Specific instructions were sent to each expert, including an explanation of the aim of the study and a description of Phases 1 and 2, which had generated the initial set of competencies and learning outcomes. Survey collection was conducted in September–October 2019. Learning outcomes were assessed on a Likert scale ranging from 0 points (completely irrelevant) to 5 points (completely relevant). The experts also evaluated the clarity and intelligibility of the wording of the competencies and learning outcomes and their adequacy to their domains and EPB steps. Based on the panel’s assessment, the competencies and learning outcomes were revised by two researchers (JD and DJ). Learning outcomes with an average score of less than 4 points were excluded.

### Ethical Issues

The study complied with the Declaration of Helsinki. Participants were informed that their consent was assumed if they responded to the survey. Anonymity and confidentiality were assured regarding use of data.

### Data Analyses

A content analysis was performed to detect and introduce new proposals or reformulations of statements. Descriptive statistics were conducted (mean, standard deviation, absolute frequency, and relative frequency) using SPSS Statistics (version 26.0).

## RESULTS

Initially, a literature review was conducted (Figure S1). A total of 21,039 records were found. Of these, 3,654 duplicates were excluded. Subsequently, 15,356 records were excluded by title, 1,727 records were excluded by abstract, and 214 were excluded by full‐text screening. The basic set of documents to be analyzed comprised 88 records from 1998 to 2018, including EBP competency reviews and consensus studies for nurses and allied healthcare professionals (Table S2). Many of the statements were constructed on the basis of previous works. Based on the content analysis of the basic set, EBP competencies for nurses were identified (*n* = 835). After EBP competency identification, statements were grouped into the seven EBP steps; duplicate and irrelevant statements were excluded (*n* = 157), as shown in Figure S2. Most statements were placed in Step 3 (25.6%), “Critically appraise the evidence that has been collected for its validity, reliability, and applicability, and then synthesize that evidence,” and Step 2 (20.3%), “Search for and collect the most relevant and best evidence to answer the clinical question.” The lowest percentage of statements was in Step 6, “Disseminate the outcomes of the EBP decision or change.” Regarding excluded statements, 29.3% were placed in Step 2 and 15.2% in Step 1.

The remaining competency statements (*n* = 678) were evaluated in the first round of Phase 2. Based on the scores of adequacy for each EBP step, 188 competency statements were retained (Figure S2). In the second round of Phase 2, competency statements were allocated to the cognitive, affective, skills, or practical domains; no agreement was reached on 45 statements, one statement was added to Step 6, and 51 statements were reformulated. The resulting subset of competency statements (*n* = 144), from round two of Phase 2, was subjected to qualitative analysis and, according to the conceptual framework adopted in the study, a final set of core EBP competencies for nurses (*n* = 24) and learning outcomes (*n* = 120) was generated for evaluation. Twenty‐three of the selected competencies were part of the EBP competency framework by Melnyk et al. (2014). Competencies 5, 6, 7, 13, 15, and 23 were minimally reformulated. Competency 16 was considered similar to competency 7 and eliminated. A new competency was added in Step 5, “Interpret obtained outcomes after the evaluation of an evidence‐based changed practice.”

Finally, in Phase 3, the set of core EBP competencies for nurses and learning outcomes was submitted for evaluation to 30 members of the international panel of experts (Table S3) identified to participate in the Delphi survey. Consensus was reached in the first round, with mean scores of all learning outcomes of 4 or higher (range 4.0–4.9), and the final set of core EBP competencies for nurses and relevant learning outcomes was established without changing the number from the previous phase. Minimal changes were suggested by the experts. In Step 3, two statements from the affective domain were reassigned, one to the cognitive domain and the other to the skills domain. Four statements were reformulated. Overall, the set included 24 competencies and 120 learning outcomes (Table S4). The distribution of competencies and learning outcomes is quite balanced, with the smallest number of listed statements in Step 1, “Ask the burning clinical question,” and the greatest number of listed statements in Step 4, “Integrate the evidence.” In the initial set of statements, Steps 2, “Search,” and 3, “Critical appraisal,” had the highest reduction in statements (around 10%) because many were redundant and received a low rating. This meant that statements in Steps 5, “Evaluate outcomes,” and 6, “Disseminate,” increased in relative proportion over the total by approximately 6–8% in the final set. The proportion of learning outcomes was 18.3% in the affective domain, 29.1% in the cognitive domain, and more than half (52.5%) in the skills domain (Figure S2).

## DISCUSSION

Nursing faculties and healthcare systems aim to ensure that healthcare professionals are competent in their clinical practice, including EBP, as recommended by international organizations and institutions (Cronenwett et al., 2007; EFN, 2015; QSEN, 2020; World Health Organization, 2017). This has led to an increased interest in establishing an EBP competency framework for nurses, mainly in the United States (Melnyk et al., 2014; Stevens, 2009) and Australia (Leung et al., 2016). Based on an updated literature review and consensus of experts from the Czech Republic, Greece, Italy, Poland, Slovenia, and Spain, 24 EBP competencies and 120 learning outcomes for general nurses and advanced practice nurses were identified. This is the first study to establish a set of EBP competencies and learning outcomes within the European nursing context.

The proposed framework has several advantages over existing ones. Our proposed competency framework is supported by a conceptual background that understands competency (Laibhen‐Parkes, 2014) as a cluster of related attitudes, knowledge, and skills that have a major impact on one’s job (role or responsibilities) and are demonstrated when a person is able to perform certain tasks within a defined context of professional practice. The reviewed statements showed two levels of specificity that, according to educational theories (Kennedy et al., 2009), permitted us to separate specific competencies and learning outcomes grouped into affective, cognitive, and skills domains. Consensus was achieved regarding the competency statements using, with minor modifications, the EBP competency framework by Melnyk et al. (2014), as previously done in a Finnish context (Saunders et al., 2019). The seven EBP steps as defined by Melnyk and Fineout‐Overholt (2015) allowed allocation of competencies and learning outcomes in a systematic way, following the EBP process, with the incorporation of two new steps compared to previous EBP competency frameworks based on the generic five‐step EBP model (Albarqouni, Hoffmann, & Glasziou, 2018; Leung et al., 2016; Straus, Glasziou, Richardson, & Haynes, 2011): Step 0, “Cultivate a spirit of inquiry within an EBP culture and environment,” and Step 6, “Disseminate the outcomes of the EBP decision or change.”

Our framework incorporates 120 learning outcomes classified into affective, cognitive, and skills domains and is associated with the most appropriate competencies and the seven‐step EBP process. Previous frameworks have used the same three domains (Cronenwett et al., 2007; QSEN, 2020) but in a less detailed manner. In our proposal, the distribution of competencies and learning outcomes is very balanced, an uncommon feature. In the aforementioned competency frameworks, the acquire and appraise steps show a greater concentration of statements (Albarqouni, Hoffmann, & Glasziou, 2018; Leung et al., 2016) and are usually overrepresented in educational strategies (Albarqouni, Hoffmann, & Glasziou, 2018). Our proposal tries to resolve this anomaly, with special attention paid to developing learning outcomes in the implementation, evaluation, and dissemination steps in more detail. In these steps, learning outcomes are mainly represented in the skills domain, highlighting its relevance to the acquisition of EBP competencies.

Nursing research conducted in Europe has been criticized as being predominantly descriptive, unnecessary, and of little relevance to clinical practice (Richards, Hanssen, & Borglin, 2018). As nurses globally account for the greatest proportion of healthcare professionals, it may be assumed that the expected EBP outcome of providing the best possible care at the lowest possible cost in a limited‐resource setting can rarely be achieved (Closs & Cheater, 1999). This will likely result in serious consequences for quality of care as well as for patient safety and outcomes (Saunders & Vehviläinen‐Julkunen, 2016). The environment of perpetual change in health care has resulted in an urgent need to educate nurses to be competent in EBP. Changes in practice must be reflected by changes in education (Oh et al., 2010).

Our study also indicated that EBP teaching varies between countries and that knowledge of EBP is integrated into bachelor’s and master’s study programs in different ways as well. EBP content is mainly included as a part of subjects (i.e., the subjects only incorporate certain aspects of EBP concepts) or as a standalone course (Skela‐Savič et al., 2020). Healthcare facilities, together with education providers, face the challenge of finding the most effective way of training and further educating nurses in EBP. One sign of a university’s effectiveness in teaching EBP is that upon completion of the EBP courses, students should be able to apply their EBP skills to practice (Zeleníková, Beach, Ren, Wolff, & Sherwood, 2014).

The set of competencies and learning outcomes was adapted and developed to respond to the specific learning needs of the European nursing discipline, as it was necessary to design new strategies for teaching EBP (Aglen, 2016). The organization of EBP into affective, cognitive, and skills domains, as suggested by Ramis et al. (2019), may serve as guidance for developing and harmonizing the content of teaching and teaching principles and strategies that may improve EBP competencies in nurses.

### Study Limitation and Future Research

The heterogeneity and differences in development between nursing levels in EBP across European countries have not facilitated differentiation between competencies and learning outcomes for general and advanced nurses. Experts from Finland found that some of the Melnyk et al. (2014) competencies for general nurses required the knowledge and skills of advanced nurses (Saunders et al., 2019). This implies the need for future research, perhaps first at national levels, to achieve consensus and to define the competency level of EBP for general and advanced nurses in Europe, and to develop a common framework to facilitate curriculum development and continuing education.

### Linking Evidence to Action

- The set of EBP competencies and learning outcomes can guide nurse educators, managers, and EBP stakeholders in the development of content that incorporates EBP knowledge, skills, and attitudes into educational programs.
- Prioritizing the EBP competencies and learning outcomes that are most necessary, and adapting them to every context, will provide healthcare organizations with guidelines for enhancing the continuing education of nurses.
- These results could facilitate the development of effective tools for assessing nursing students’ and nurses’ perception of competencies required for EBP processes.

## CONCLUSIONS

Based on a literature review and expert consensus, a set of core EBP competencies for nurses and the most important EBP learning outcomes was developed for implementation in nursing education programs across European countries.

## Supporting information

Figure S1 PRISMA flow diagram (Moher et al., 2009).

Table S1 Data Extraction Protocol

Table S2 Characteristics of Included Studies
Figure S2 Sequence of selection and distribution of statements along the three phases of the study.
Table S3 Characteristics of the Delphi Survey Participants
Table S4 Set of Core EBP Competencies and Relevant Learning Outcomes for General and Advanced Nurses
