# Supplementary material for: Core Evidence‐Based Practice Competencies and Learning Outcomes for European Nurses: Consensus Statements
Source: Worldviews Evid Based Nurs. 2021 May 24;18(3):226–33. doi: 10.1111/wvn.12506 (PMC8251814; doi:10.1111/wvn.12506)
Supplement: Supplementary file 1 — Figure S1 PRISMA flow diagram (Moher et al., 2009). [file WVN-18-226-s003.docx]

**Figure S1.** PRISMA flow diagram (Moher et al., 2009).

Studies included in qualitative synthesis
(*n* = 88)

Records excluded by abstract
(*n* = 1,727)

Records excluded by title
(*n* = 15,356)

Duplicates excluded
 (*n* = 3,654)

Records for title review
(*n* = 17,385)

Records for abstract review
(*n* = 2,029)

Full-text articles assessed for eligibility
(*n* = 302)

Total number of records for title review (*n* = 21,039)

Full-text articles excluded, with reasons
(*n* = 214)

**International databases** (*n* = 17,158)

ProQuest (*n* = 274) EBSCO (*n* = 1,734)

Web of Science (*n* = 845) SpringerLink (*n* = 4,728)

CINAHL (*n* = 317) ScienceDirect (*n* = 2,937)

Cochrane Library (*n* = 133) SCOPUS (*n* = 3,031)

PubMed (*n* = 2,609) PsycINFO (*n* = 283)

EMBASE (*n* = 268)

**National databases** (*n* = 3,881)
