# Supplementary material for: Core Evidence‐Based Practice Competencies and Learning Outcomes for European Nurses: Consensus Statements
Source: Worldviews Evid Based Nurs. 2021 May 24;18(3):226–33. doi: 10.1111/wvn.12506 (PMC8251814; doi:10.1111/wvn.12506)
Supplement: Supplementary file 2 — Table S1 Data Extraction Protocol [file WVN-18-226-s002.docx]

| **Data Extraction Protocol**  **Activity dates:**  **Start 2^nd^ January 2019**  **End 28^th^ February 2019** |
| --- |

| **Title of review** |
| --- |
| Definition of EBP competencies for general and advanced nurses |

| **Objective** |
| --- |
| Identify and analyze studies focusing on EBP competencies for general and advanced nurses |

| **Review questions** |
| --- |
| What are core EBP competencies for general and advanced nurses? |

| **Keywords in alphabetical order** |
| --- |
| Competence; Curriculum; Evidence-based nursing; Evidence-based practice; Learning; Nurse; Nursing degree; Skills |

| **Research limits** | |
| --- | --- |
| Language | English and languages of the participants  (Spanish, Polish, Italian, Greek, Slovenian, and Czech) |
| Year of publication | 1998–2018 |
| Type of documents | Observational, quasi-experimental, and experimental studies, reviews, reports, and guidelines |

| **Selection criteria** | |
| --- | --- |
| Inclusion criteria | Studies focusing on EBP competencies for nurses and other healthcare (HC) professionals  *Note.* We must collect the competencies separately between nurses and other HC professionals. Many of these competencies are similar to other HC professionals. |
| Exclusion criteria | None at this moment (may include exclusion criteria after the initial review) |

| **Core bibliographic databases** | |
| --- | --- |
| International databases | CINAHL Plus with Full Text |
|  | SpringerLink |
|  | Cochrane Library |
|  | ProQuest |
|  | ScienceDirect |
|  | Web of Knowledge |
|  | SCOPUS |
|  | EBSCO |
|  | PubMed |
|  | EMBASE |
|  | PsycINFO |
|  | Others (each team could add other international databases that they will use in their searches) |
| National databases | Bibliographic databases of Spanish, Polish, Italian, Greek, Slovenian, and Czech |
| Other resources | Associations, institutions’ webpages, books, theses |

| **Search operator** |
| --- |
| Boolean operators – binary operator (AND, OR) |

| **Search terms** | |
| --- | --- |
| 1. | (evidence-based practice OR evidence-based nursing OR EBP OR EBN) AND (competence OR skills) AND nurse |
| 2. | (evidence-based practice OR evidence-based nursing OR EBP OR EBN) AND curriculum AND nursing degree AND (learning OR teaching) |

| **Data storage** |
| --- |
| For data storage use Mendeley. That will permit storage and to share the records. You can download the app for your computer from [www.mendeley.com](http://www.mendeley.com). It is free, and you can create a desk version that is very easy to manage. |

| **Data extraction** |
| --- |
| For data extraction use PRISMA flow diagram:  <http://prisma-statement.org/prismastatement/flowdiagram.aspx> |

| **Data evaluation** | |
| --- | --- |
| Analysis of selected data | Description of the records in a table showing the next information (enter in DRIVE and include your data in the file “Competencies on EBP Systematic Review.” Each team has to use its own country sheet: Complete reference, language, type of document, author, year of publication, title, type of study, geographical location, objectives, methods, measure, sample, results, set of competencies specifically for nurses and set of competencies for other healthcare professions, conclusion |

| **Final report** | |
| --- | --- |
| Set of competencies | Final report including a set of competencies selected after the review. It is important to include a list of the competencies in English. Avoid duplicate competencies.  *Note.* We must collect the competencies separately between nurses and other HC professionals. Many of these competencies are similar to other HC professionals. |

**Annex I**

You have to indicate the next information regarding your search. Repeat this annex for each database where you search:

1. Database name

2. Search date

3. Searching terms used (at the protocol we have the English searching terms, but maybe this is not adequate for your own language database, and you have to modify the terms, which is not a problem but you always have to clarify the searching terms)

4. Limits included if appropriate

5. Results (number of registers)

6. Selected registers (after reading the title and abstract we have to indicate the number of registers that we are going to select for reading full text)

Finally, we have to extract the data of the selected register from the Excel file that you will find in DRIVE and include your data in the file “Competencies on EBP Systematic Review.” Each team has to use its own country sheet.
