# Supplementary material for: Core Evidence‐Based Practice Competencies and Learning Outcomes for European Nurses: Consensus Statements
Source: Worldviews Evid Based Nurs. 2021 May 24;18(3):226–33. doi: 10.1111/wvn.12506 (PMC8251814; doi:10.1111/wvn.12506)
Supplement: Supplementary file 3 — Table S2 Characteristics of Included Studies Figure S2 Sequence of selection and distribution of statements along the three phases of the study. Table S3 Characteristics of the Delphi Survey Participants Table S4 Set of Core EBP Competencies and Relevant Learning Outcomes for General and Advanced Nurses [file WVN-18-226-s001.docx]

**Table S2.** Characteristics of Included Studies

| Author(s) | Year of publication | Title of the document | DOI of article |
| --- | --- | --- | --- |
| French B | 1998 | Developing skills for evidence-based practice | - 10.1016/s0260-6917(98)80034-8 |
| Johnson JM, Leung GM, Fielding R, Tin KY, Lai-Mong Ho | 2003 | The development and validation of a knowledge, attitude and behaviour questionnaire to assess undergraduate evidence-based practice teaching and learning | - 10.1046/j.1365-2923.2003.01678.x |
| McNeil BJ, Elfrink VL, Bickford CJ, Pierce ST, Beyea SC, Averill C, Klappenbach C | 2003 | Nursing Information Technology Knowledge, Skills, and Preparation of Student Nurses, Nursing Faculty, and Clinicians: A U.S. Survey | not specified |
| Burke LE, Schlenk EA, Sereika SM, Cohen SM, Happ MB, Dorman JS | 2005 | Developing Research Competence to Support Evidence-Based Practice | - [10.1016/j.profnurs.2005.10.011](https://doi.org/10.1016/j.profnurs.2005.10.011) |
| Callister LC, Matsumura G, Lookinland S, Mangum S, Loucks C | 2005 | Inquiry in Baccalaureate Nursing Education: Fostering Evidence-Based Practice | not specified |
| Pierce ST | 2005 | Integrating Evidence-Based Practice into Nursing Curricula | not specified |
| Thompson C, McCaughan D, Cullum N, Sheldon T, Raynor P | 2005 | Barriers to Evidence-Based Practice in Primary Care Nursing—Why Viewing Decision-Making as Context Is Helpful | - [10.1111/j.1365-2648.2005.03609.x](https://doi.org/10.1111/j.1365-2648.2005.03609.x) |
| Upton D, Upton P | 2006 | Development of an Evidence-Based Practice Questionnaire for Nurses | - [10.1111/j.1365-2648.2006.03739.x](https://doi.org/10.1111/j.1365-2648.2006.03739.x) |
| Gerrish K, Ashworth P, Lacey A, Bailey J, Cooke J, Kendall S, McNeilly E | 2007 | Factors influencing the development of evidence-based practice: A research tool | - [10.1111/j.1365-2648.2006.04112.x](https://doi.org/10.1111/j.1365-2648.2006.04112.x) |
| Sheriff KL, Wallis M, Chaboy W | 2007 | Nurses' Attitudes to and Perceptions of Knowledge and Skills Regarding Evidence-Based Practice | - [10.1111/j.1440-172X.2007.00651.x](https://doi.org/10.1111/j.1440-172x.2007.00651.x) |
| Emerson RJ, Records K | 2008 | Today's challenge, tomorrow's excellence: The practice of evidence-based education | - [10.3928/01484834-20080801-04](https://doi.org/10.3928/01484834-20080801-04) |
| Hart P, Eaton LA, Buckner M, Morrow BN, Barrett DT, Fraser DD, Hooks D, Sharrer RL | 2008 | Effectiveness of a Computer-Based Educational Program on Nurses’ Knowledge, Attitude, and Skill Level Related to Evidence-Based Practice | - [10.1111/j.1741-6787.2008.00123.x](https://doi.org/10.1111/j.1741-6787.2008.00123.x) |
| Kring DL | 2008 | Clinical nurse specialist practice domains and evidence-based practice competencies: A matrix of influence | - [10.1097/01.NUR.0000311706.38404.cf](https://doi.org/10.1097/01.nur.0000311706.38404.cf) |
| Meeker MA, Jones JM, Flanagan NA | 2008 | Teaching undergraduate nursing research from an evidence-based practice perspective | - [10.3928/01484834-20080801-06](https://doi.org/10.3928/01484834-20080801-06) |
| Munroe D, Duffy P, Fisher Ch | 2008 | Nurse Knowledge, Skills, and Attitudes Related to Evidence-Based Practice: Before and After Organizational Supports | not specified |
| Schmidt NA | 2008 | Evidence-Based Practice in the Nursing Curriculum: Ponderings on Design and Implementation | not specified |
| Singleton J, Levin R | 2008 | Strategies for Learning Evidence-Based Practice: Critically Appraising Clinical Practice Guidelines | - [10.3928/01484834-20080801-07](https://doi.org/10.3928/01484834-20080801-07) |
| Smith-Strøm H, Nortvedt MW | 2008 | Evaluation of Evidence-Based Methods Used to Teach Nursing Students to Critically Appraise Evidence | - [10.3928/01484834-20080801-08](https://doi.org/10.3928/01484834-20080801-08) |
| Bradley D, Dixon JF | 2009 | Staff nurses creating safe passage with evidence-based practice | - [10.1016/j.cnur.2008.10.002](https://doi.org/10.1016/j.cnur.2008.10.002) |
| Brown CE, Kim SC, Stichler JF, Fields W | 2009 | Predictors of knowledge, attitudes, use and future use of evidence-based practice among baccalaureate nursing students at two universities | - [10.1016/j.nedt.2009.10.021](https://doi.org/10.1016/j.nedt.2009.10.021) |
| Hockenberry M, Brown T, Walden M, Barrera P | 2009 | Teaching Evidence-Based Practice Skills in a Hospital | - [10.3928/00220124-20090101-08](https://doi.org/10.3928/00220124-20090101-08) |
| Ilic D | 2009 | Assessing competency in Evidence Based Practice: Strengths and limitations of current tools in practice | - [10.1186/1472-6920-9-53](https://doi.org/10.1186/1472-6920-9-53) |
| Patterson R, Carter-Templeton H, Russell C | 2009 | Information Literacy: Using LISTEN Project Strategies to Equip Nurses Worldwide | not specified |
| Waters D, Crisp J, Rychetnik L, Barratt A | 2009 | Views on evidence from nursing and midwifery opinion leaders | - [10.1016/j.nedt.2009.04.006](https://doi.org/10.1016/j.nedt.2009.04.006) |
| Geum OE, Sunah K, So Sun K, Sue K, Eun Yong Ch et al. | 2010 | Integrating Evidence-Based Practice into RN-to-BSN Clinical Nursing Education | - [10.3928/01484834-20100331-02](https://doi.org/10.3928/01484834-20100331-02) |
| Missal B, Schafer BK, Halm MA, Schaffer MA | 2010 | A University and Health Care Organization Partnership to Prepare Nurses for Evidence-Based Practice | - [10.3928/01484834-20100430-06](https://doi.org/10.3928/01484834-20100430-06) |
| Foo S, Majid S, Mokhtar IA, Zhang X, Luyt B, Chang YK, Theng YL | 2011 | Nurses’ Perception of Evidence-Based Practice at the National University Hospital of Singapore | - [10.3928/00220124-20110516-04](https://doi.org/10.3928/00220124-20110516-04) |
| Gerrish K, Guillaume L, Kirshbaum M, McDonnell A, Tod A, Nolan M | 2011 | Factors influencing the contribution of advanced practice nurses to promoting evidence‐based practice among front‐line nurses: Findings from a cross‐sectional survey | - [10.1111/j.1365-2648.2010.05560.x](https://doi.org/10.1111/j.1365-2648.2010.05560.x) |
| Dalheim A, Harthug S, Nilsen RM, Nortvedt MW | 2012 | Factors influencing the development of evidence-based practice among nurses: A self-report survey | - [10.1186/1472-6963-12-367](https://doi.org/10.1186/1472-6963-12-367) |
| Hagler D, Mays MZ, Stillwell SB, Kastenbaum B, Brooks R, Fineout-Overholt E, Williamson KM, Jirsak J | 2012 | Preparing Clinical Preceptors to Support Nursing Students in Evidence-Based Practice | - [10.3928/00220124-20120815-27](https://doi.org/10.3928/00220124-20120815-27) |
| Mokhtar IA, Majid S, Foo S, Zhang X, Theng YL, Chang YK, Luyt B | 2012 | Evidence-based Practice and Related Information Literacy Skills of Nurses in Singapore: An Exploratory Case Study | - [10.1177/1460458211434753](https://doi.org/10.1177/1460458211434753) |
| Mollon D, Fields W, Gallo AM, Wagener R, Soucy J, Gustafson B, Kim SC | 2012 | Staff Practice, Attitudes, and Knowledge/Skills Regarding Evidence-Based Practice Before and After an Educational Intervention | - [10.3928/00220124-20120716-89](https://doi.org/10.3928/00220124-20120716-89) |
| Rudman A, Gustavsson P, Ehrenberg A, Boström AM, Wallin L | 2012 | Registered Nurses' Evidence-Based Practice: A Longitudinal Study of the First Five Years After Graduation | - [10.1016/j.ijnurstu.2012.07.007](https://doi.org/10.1016/j.ijnurstu.2012.07.007) |
| Youn-Jung S, Sun-Hee K, Young-Su P, Soo-Kyoung L, Yun L | 2012 | The Influence of Information Retrieval Skill on Evidence Based Practice Competency in Clinical Nurses | 10.7475/kjan.2012.24.6.635 |
| Finotto S, Carpanoni M, Turroni EC, Camellini R, Mecugni D | 2013 | Teaching evidence-based practice: Developing a curriculum model to foster evidence-based practice in undergraduate student nurses | - [10.1016/j.nepr.2013.03.021](https://doi.org/10.1016/j.nepr.2013.03.021) |
| Gerrish K, Cooke J | 2013 | Factors influencing evidence-based practice among community nurses | - [10.1111/j.1365-2648.2006.04112.x](https://doi.org/10.1111/j.1365-2648.2006.04112.x) |
| Heiwe S, Johansson E, Nilsson-Kajermo K, Säflund K, Olin AO | 2013 | Outcomes of a Multiprofessional Educational Intervention in Evidence-Based Practice | not specified |
| Ruzafa-Martinez M, Lopez-Iborra L, Moreno-Casbas T, Madrigal-Torres M | 2013 | Development and validation of the competence in evidence based practice questionnaire (EBP-COQ) among nursing students | - [10.1186/1472-6920-13-19](https://doi.org/10.1186/1472-6920-13-19) |
| Thorsteinsson HS | 2013 | Icelandic Nurses’ Beliefs, Skills, and Resources Associated with Evidence-Based Practice and Related Factors: A National Survey | - [10.1111/j.1741-6787.2012.00260.x](https://doi.org/10.1111/j.1741-6787.2012.00260.x) |
| Ammouri AA, Raddaha AA, Dsouza P, Geethakrishnan R, Noronha JA, Obeidat AA, Shakman L | 2014 | Evidence-Based Practice: Knowledge, Attitudes, Practice and Perceived Barriers Among Nurses in Oman | not specified |
| Laibhen-Parkes N | 2014 | Evidence-Based Practice Competence: A Concept Analysis | - [10.1111/2047-3095.12035](https://doi.org/10.1111/2047-3095.12035) |
| Leung K, Trevena L, Waters D | 2014 | Systematic review of instrument for measuring nurses knowledge, skills and attitudes for evidence-based practice | - [10.1111/jan.12454](https://doi.org/10.1111/jan.12454) |
| Melnyk BM, Gallagher-Ford L, Long LE, Fineout-Overholt E | 2014 | The Establishment of Evidence-Based Practice Competencies for Practicing Registered Nurses and Advanced Practice Nurses in Real-World Clinical Settings: Proficiencies to Improve Healthcare Quality, Reliability, Patient Outcomes, and Costs | - [10.1111/wvn.12021](https://doi.org/10.1111/wvn.12021) |
| Stokke K, Olsen NR, Espehaug B, Nortvedt MW | 2014 | Evidence based practice beliefs and implementation among nurses: A cross-sectional study | - [10.1186/1472-6955-13-8](https://doi.org/10.1186/1472-6955-13-8) |
| Thorsteinsson HS, Sveinsdóttir H | 2014 | Readiness for and Predictors of Evidence-Based Practice of Acute-Care Nurses: A Cross-Sectional Postal Survey | - [10.1111/scs.12083](https://doi.org/10.1111/scs.12083) |
| Zelenikova R, Jarosová D | 2014 | Perception of the effectiveness of evidence-based practice courses by Czech nursing and midwifery students | 10.15452/CEJNM.2014.05.0013 |
| Dotson BJ, Lewis LS, Aucoin JW, Murray S, Chapin D, Walters P | 2015 | Teaching evidence-based practice (EBP) across a four-semester nursing curriculum | not specified |
| Farokhzadian J, Khajouei R, Ahmadian L | 2015 | Evaluating factors associated with implementing evidence-based practice in nursing | - [10.1111/jep.12480](https://doi.org/10.1111/jep.12480) |
| Karki S, Acharya R, Budhwani H, Shrestha P, Chalise P, Shrestha U, Gautam K, Wilson L | 2015 | Perceptions and Attitudes towards Evidence Based Practice among Nurses and Nursing Students in Nepal | - [10.3126/kumj.v13i4.16829](https://doi.org/10.3126/kumj.v13i4.16829) |
| Melnyk BM, Gallagher‐Ford L | 2015 | Implementing the New Essential Evidence‐Based Practice Competencies in Real‐world Clinical and Academic Settings: Moving From Evidence to Action in Improving Healthcare Quality and Patient Outcomes | - [10.1111/wvn.12089](https://doi.org/10.1111/wvn.12089) |
| Ock GM, Yeongmi H, Jeong Sook K | 2015 | Development and validation of an instrument to assess knowledge and skills of evidence-based nursing | [10.1111/jocn.12754](https://doi.org/10.1111/jocn.12754) |
| Phelps SF, Hyde L, Wolf JP | 2015 | Introducing Information Literacy Competency Standards for Nursing | - [10.1097/NNE.0000000000000170](https://doi.org/10.1097/nne.0000000000000170) |
| Ramos-Morcillo AJ, Fernandez-Salazar S, Ruzafa-Martinez M, Del-Pino-Casado R | 2015 | Effectiveness of a Brief, Basic Evidence-Based Practice Course for Clinical Nurses | - [10.1111/wvn.12103](https://doi.org/10.1111/wvn.12103) |
| Yeon-Sook K, Jimee K, Mi-Mi P | 2015 | Factors Influencing Competency in Evidence-based Practice among Clinical Nurses | [10.11111/jkana.2015.21.2.143](https://doi.org/10.11111/jkana.2015.21.2.143) |
| Bissett KM, Cvach M, White KM | 2016 | Improving Competence and Confidence With Evidence-Based Practice Among Nurses: Outcomes of a Quality Improvement Project | - [10.1097/NND.0000000000000293](https://doi.org/10.1097/nnd.0000000000000293) |
| Bostwick L, Linden L | 2016 | Evaluation Criteria for Nursing Student Application of Evidence-Based Practice: A Delphi Study | - [10.3928/01484834-20160516-06](https://doi.org/10.3928/01484834-20160516-06) |
| Davidson SJ, Candy L | 2016 | Teaching EBP Using Game-Based Learning: Improving the Student Experience | - [10.1111/wvn.12152](https://doi.org/10.1111/wvn.12152) |
| Ehrenberg A, Gustavsson P, Wallin L, Bostrom AM, Rudman A | 2016 | New Graduate Nurses’ Developmental Trajectories for Capability Beliefs Concerning Core Competencies for Healthcare Professionals: A National Cohort Study on Patient-Centered Care, Teamwork, and Evidence-based Practice | - [10.1111/wvn.12178](https://doi.org/10.1111/wvn.12178) |
| Leung K, Trevena L, Waters D | 2016 | Development of a competency framework for evidence-based practice in nursing | - [10.1016/j.nedt.2016.01.026](https://doi.org/10.1016/j.nedt.2016.01.026) |
| Malik G, McKenna L, Griffiths D | 2016 | Envisaging the use of evidence-based practice (EBP): How nurse academics facilitate EBP use in theory and practice across Australian undergraduate programmes | - [10.1111/jocn.13705](https://doi.org/10.1111/jocn.13705) |
| Mallion J, Brooke J | 2016 | Community- and hospital-based nurses’ implementation of evidence-based practice: Are there any differences? | - [10.12968/bjcn.2016.21.3.148](https://doi.org/10.12968/bjcn.2016.21.3.148) |
| Orta R, Messmer PR, Valdes GR, Turkel M, Fields SD, Wei ChC | 2016 | Knowledge and Competency of Nursing Faculty Regarding Evidence-Based Practice | - [10.3928/00220124-20160817-08](https://doi.org/10.3928/00220124-20160817-08) |
| Zabaleta‐del‐Olmo E, Subirana‐Casacuberta M, Ara‐Pérez A, Escuredo‐Rodríguez B, Ríos‐Rodríguez MÁ, Carrés‐Esteve L, Ondiviela‐Cariteu À | 2016 | Developing Evidence-Based Practice Questionnaire for Community Health Nurses: Reliability and Validity of a Spanish Adaptation | - [10.1111/jocn.13078](https://doi.org/10.1111/jocn.13078) |
| Azar FE, Rezapour A, Isfahani HM, Azami-Aghdash S, Kalavani K, Mahmoudi F | 2017 | Evidence-based medicine performance among health care providers in Iranian hospitals: A nationwide survey | - [10.14196/mjiri.31.77](https://doi.org/10.14196/mjiri.31.77) |
| Hamaideh SH | 2017 | Sources of Knowledge and Barriers of Implementing Evidence-Based Practice Among Mental Health Nurses in Saudi Arabia | - [10.1111/ppc.12156](https://doi.org/10.1111/ppc.12156) |
| Hande K, CuT W, Robbins HM, Kennedy BB, Christenbury T | 2017 | Leveling Evidence-based Practice Across the Nursing Curriculum | not specified |
| Moore L | 2017 | Effectiveness of an Online Educational Module in Improving Evidence-Based Practice Skills of Practicing Registered Nurses | - [10.1111/wvn.12214](https://doi.org/10.1111/wvn.12214) |
| Mulenga Ch, Naidoo JR | 2017 | Nurses’ knowledge, attitudes and practices regarding evidence-based practice in the prevention of mother-to-child transmission of HIV programme in Malawi | - [10.4102/curationis.v40i1.1656](https://doi.org/10.4102/curationis.v40i1.1656) |
| Patelarou AE, Katsouli K, Stamou A, Vivilaki V, Koukia E, Sifaki-Pistolla D, Patelarou E | 2017 | Attitudes, knowledge and perceptions of psychiatric nurses about evidence-based practice | not specified |
| Reid J, Briggs J, Carlisle S, Scott D, Lewis C | 2017 | Enhancing utility and understanding of evidence based practice through undergraduate nurse education | - [10.1186/s12912-017-0251-1](https://doi.org/10.1186/s12912-017-0251-1) |
| Rojjanasrirat W, Rice J | 2017 | Evidence-based practice knowledge, attitudes, and practice of online graduate nursing students | - [10.1016/j.nedt.2017.04.005](https://doi.org/10.1016/j.nedt.2017.04.005) |
| Skela-Savič B, Hvalič-Touzery S, Pesjak K | 2017 | Professional Values and Competencies as Explanatory Factors for the Use of Evidence-Based Practice in Nursing | - [10.1111/jan.13280](https://doi.org/10.1111/jan.13280) |
| Spiva LA, Hart PL, Patrick S, Waggoner J, Jackson Ch, Threatt JT | 2017 | Effectiveness of an Evidence-Based Practice Nurse Mentor Training Program | - [10.1111/wvn.12219](https://doi.org/10.1111/wvn.12219) |
| Thomas A, Han L, Osler BP, Turnbull EA, Douglas E | 2017 | Students’ attitudes and perceptions of teaching and assessment of evidence based practice in an occupational therapy professional Master’s curriculum: A mixed methods study | [10.1186/s12909-017-0895-2](https://dx.doi.org/10.1186%2Fs12909-017-0895-2) |
| Albarqouni L, Hoffmann T, Straus S, Olsen NR, Young T, Ilic D, et al. | 2018 | Core Competencies in Evidence-Based Practice for Health Professionals: Consensus Statement Based on a Systematic Review and Delphi Survey | - [10.1001/jamanetworkopen.2018.0281](https://doi.org/10.1001/jamanetworkopen.2018.0281) |
| Arechabala MC, Salamanca MIC, Silva NR, Acuña MR, Aldunate PC | 2018 | Implementación de la práctica basada en evidencia en el currículo de Enfermería/Implementation of evidence based practice in the nursing curriculum | not specified |
| Cuia Ch, Lia Y, Genb D, Zhang H, Zinc Ch | 2018 | The effectiveness of evidence-based nursing on development of nursing students' critical thinking: A meta-analysis | - [10.1016/j.nedt.2018.02.036](https://doi.org/10.1016/j.nedt.2018.02.036) |
| Goota WE, Keersa JC, Kuipersa R, Niewegb RMB, Grootc M | 2018 | The effect of a multifaceted evidence-based practice programme for nurses on knowledge, skills, attitudes, and perceived barriers: A cohort study | - [10.1016/j.nedt.2018.01.008](https://doi.org/10.1016/j.nedt.2018.01.008) |
| Hannele S, Vehvilainen-Julkunen K | 2018 | Key considerations for selecting instruments when evaluating healthcare professionals’ evidence‐based practice competencies: A discussion paper | - [10.1111/jan.13802](https://doi.org/10.1111/jan.13802) |
| Horntvedt MT, Nordsteien A, Fermann T, Severinsson E | 2018 | Strategies for teaching evidence-based practice in nursing education: A thematic literature review | - [10.1186/s12909-018-1278-z](https://doi.org/10.1186/s12909-018-1278-z) |
| Hsieh PL, Chen SH, Chang LC | 2018 | School Nurses’ Perceptions, Knowledge, and Related Factors Associated with Evidence-Based Practice in Taiwan | - [10.3390/ijerph15091845](https://doi.org/10.3390/ijerph15091845) |
| Laibhen-Parkes N, Kimble LP, Melnyk BM, Sudia T, Codone S | 2018 | An Adaptation of the Original Fresno Test to Measure Evidence-Based Practice Competence in Pediatric Bedside Nurses | - [10.1111/wvn.12289](https://doi.org/10.1111/wvn.12289) |
| Melnyk BM | 2018 | Breaking Down Silos and Making Use of the Evidence‐Based Practice Competencies in Healthcare and Academic Programs: An Urgent Call to Action | - [10.1111/wvn.12271](https://doi.org/10.1111/wvn.12271) |
| Melnyk BM, Gallagher‐Ford L, Zellefrow C, Tucker S, Thomas B, Sinnott LT, Tan A | 2018 | The First U.S. Study on Nurses’ Evidence-Based Practice Competencies Indicates Major Deficits That Threaten Healthcare Quality, Safety, and Patient Outcomes | - [10.1111/wvn.12269](https://doi.org/10.1111/wvn.12269) |
| Saunders H, Vehviläinen-Julkunen K | 2018 | Key considerations for selecting instruments when evaluating healthcare professionals’evidence‐based practice competencies: A discussion paper | - [10.1111/jan.13802](https://doi.org/10.1111/jan.13802) |
| Schuman CJ, Ploutz-Snyder RJ, Titler MG | 2018 | Development and Testing of the Nurse Manager EBP Competency Scale | - [10.1177/0193945917728249](https://doi.org/10.1177/0193945917728249) |
| Yonkaitis CF | 2018 | Evidence-Based Practice and School Nurse Practice: A Review of Literature | - [10.1177/1059840517728108](https://doi.org/10.1177/1059840517728108) |
| Youssefa NFA, Alshraifeenb A, Alnuaimic K, Uptond P | 2018 | Egyptian and Jordanian nurse educators' perception of barriers preventing the implementation of evidence-based practice: A cross-sectional study | - [10.1016/j.nedt.2018.01.035](https://doi.org/10.1016/j.nedt.2018.01.035) |

**Figure S2.** Sequence of selection and distribution of statements along the three phases of the study.


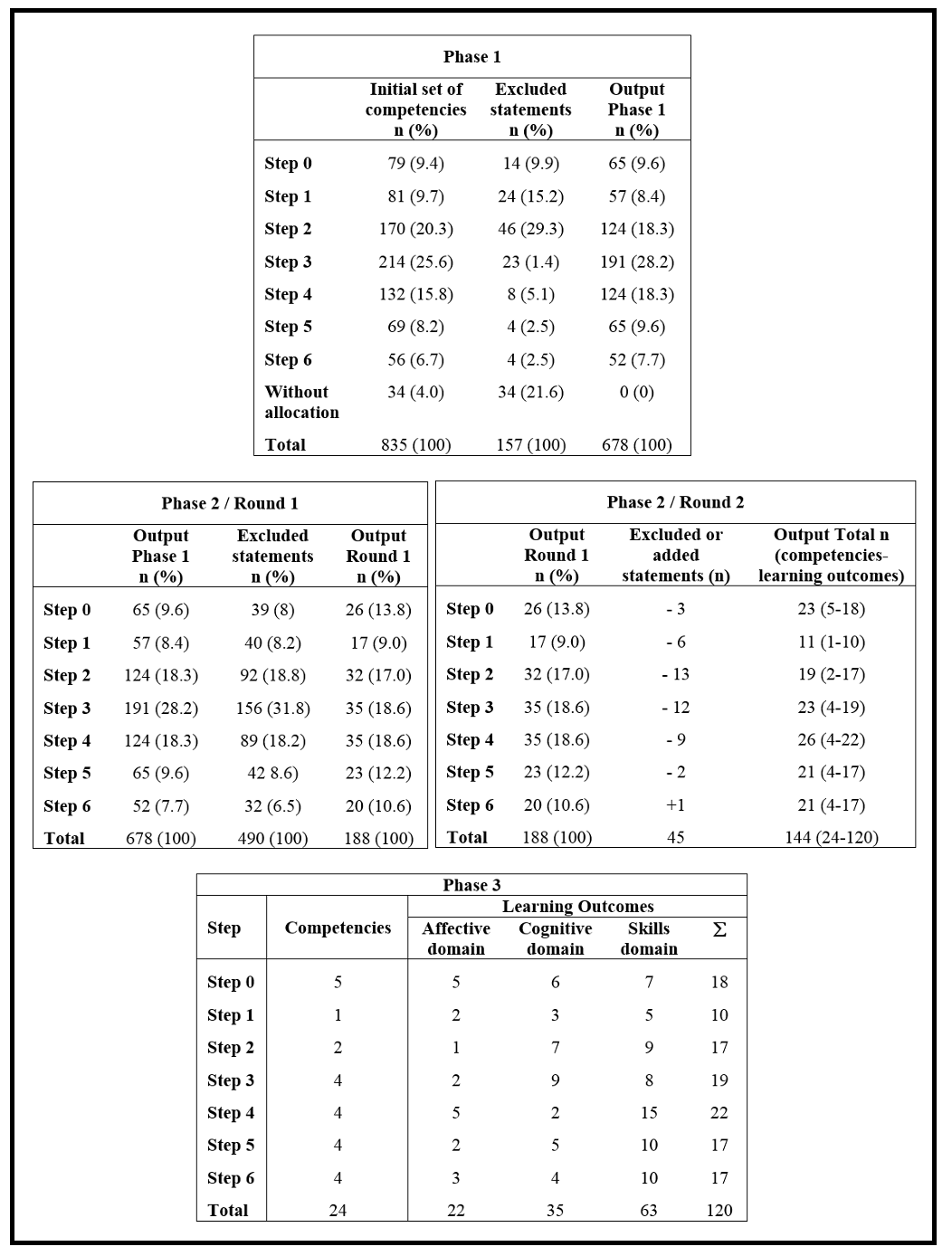


**Table S3.** Characteristics of the Delphi Survey Participants

| **Characteristic** | **Mean (SD)** |
| --- | --- |
| **Age, years** | 47.8 (8.4) |
| **EBP teaching experience, years** | 8.1 (4.2) |
| **Clinical experience, years** | 13.7 (8.1) |
|  | ***n* (%)** |
| **Age, years** |  |
| < 30 | 0 (0) |
| 30-44 | 11 (36.7) |
| 45-59 | 17 (56.6) |
| ≥ 60 | 2 (6.7) |
| **Sex** | |
| Female | 25 (83.3) |
| Male | 5 (16.7) |
| **Countries** |  |
| Czech Republic | 6 (20) |
| Greece | 5 (16.6) |
| Italy | 5 (16.6) |
| Poland | 4 (13.4) |
| Slovenia | 4 (13.4) |
| Spain | 6 (20) |
| **Current role** | |
| Academic | 28 (93.4) |
| Clinical | 1 (3.3) |
| Research | 1 (3.3) |
| **Setting or institution** | |
| University | 27 (90.1) |
| Hospital | 0 (0) |
| Other | 3 (9.9) |
| **Currently teaching EBP** | |
| Yes | 21 (70.1) |
| No | 9 (29.9) |

*Note.* SD = standard deviation

**Table S4.** Set of Core EBP Competencies and Relevant Learning Outcomes for General and Advanced Nurses

| **STEP 0. CULTIVATE A SPIRIT OF INQUIRY WITHIN AN EBP CULTURE AND ENVIRONMENT** | | | | |
| --- | --- | --- | --- | --- |
| **Competencies** | **Learning outcomes related to EBP competency for nursing roles** | | | |
| - Questions clinical practices for the purpose of improving the quality of care. - Describes clinical problems using internal evidence (evidence generated internally within a clinical setting, such as patient assessment data, outcomes management, and quality improvement data). - Participates to establish and sustain an evidence-based practice culture. - Mentors others in evidence-based decision-making and the EPB process. - Implements strategies to establish and sustain an EBP culture. | *Affective domain* | - Assumes the role of a change agent for the organization. - Believes that EBP results are the best clinical care for patients. - Fosters EBP organizational culture, infrastructure, and teamwork. - Supports a culture of inquiry. - Understands the impact of the clinical practice questioning on improving individual/group health outcomes. | |  |
|  | *Cognitive domain* | - Describes ethical principles related to variation in practice and EBP. - Explains the purpose and importance of EBP in clinical practice. - Explains the significance of practice variation related to evidence-based care. - Justifies that seven steps of EBP help to make decisions in clinical practice. - Defines EBP as the integration of the best research evidence with clinical expertise and the patient’s unique values and circumstances. - Understands the distinction between using research to inform clinical decision-making vs. conducting research. | |  |
|  | *Skills domain* | - Identifies gaps in the clinical practice. - Identifies the need for change based on evidence. - Acts as a resource on current, rapidly advancing evidence-based quality initiatives and change forces. - Addresses clinical problems and quality improvement issues with the evidence-based practice process. - Participates in the organizational culture of evidence-based quality improvement in care. - Provides leadership for integrating EBP in clinical practice. - Facilitates movement of practice change through formal institutional channels collaborating with stakeholders and resource managers. | |  |
| **STEP 1. ASK THE BURNING CLINICAL QUESTION IN THE FORMAT THAT WILL YIELD THE MOST RELEVANT AND BEST EVIDENCE (I.E., PICOT FORMAT)** | | | | |
| **Competencies** | **Learning outcomes related to EBP competency for nursing roles** | | | |
| - Converts a clinical problem into an answerable clinical question using a structured format (i.e., PICO(T) or others). | *Affective domain* | | - Recognizes the relevance of meaningful clinical questions to address clinical practice. - Keeps an open mind to be an inquiry about the clinical practice. |  |
|  | *Cognitive domain* | | - Differentiates the key components of a structured clinical question (PICO(T) format or others). - Explains the difference between the types of questions that cannot typically be answered by research (background questions) and those that can (foreground questions). - Identifies different types of clinical questions, such as questions about treatment, diagnosis, prognosis, etiology, and meaning. |  |
|  | *Skills domain* | | - Formulates a structured question in response to clinical query/issue. - Classifies the major study designs for each type of clinical question. - Identifies clinical problems that can be addressed through evidence-based practice. - Uses frequency and relevance criteria to prioritize clinical questions. - Uses generic accepted terminology in creating a structured clinical question. |  |
| **STEP 2. SEARCH FOR AND COLLECT THE MOST RELEVANT AND BEST EVIDENCE TO ANSWER THE CLINICAL QUESTION (E.G., SEARCHING FOR SYSTEMATIC REVIEWS, INCLUDING META-ANALYSES)** | | | | |
| **Competencies** | **Learning outcomes related to EBP competency for nursing roles** | | | |
| - Searches for external evidence (evidence generated from research) to answer focused clinical questions. - Systematically conducts an exhaustive search for external evidence to answer a clinical question | *Affective domain* | | - Is aware that it is necessary to screen and select the appropriate information to answer a clinical question. |  |
|  | *Cognitive domain* | | - Recognizes major document types and resources included on principal bibliographic databases. - Classifies scientific evidence as primary research evidence, synthesis (systematic reviews), or evidence summary. - Describes the hierarchy search of a clinical question. - Describes the principal health sciences and allied sciences bibliographic databases/resources to search. - Details the search strategies (controls vocabulary, thesaurus, keywords, limit function, and Boolean operators). - Distinguishes scientific evidence as primary research evidence and pre-appraised literature (systematic reviews, clinical practice guidelines, evidence summaries, etc.). - Distinguishes between filtered (pre-appraised) and unfiltered (un-appraised) database resources and recognizes the common databases in use (e.g., Medline, CINHAL, etc.). |  |
|  | *Skills domain* | | - Conducts a systematic approach to search for evidence in appropriate databases (i.e., development of synonyms from the PICO question and correct use of Boolean operators per the database in a search). - Searches the literature on electronic databases and online repositories. - Constructs an appropriate search strategy for answering clinical questions. - Reviews and selects the appropriate evidence after reading the title and abstract. - Demonstrates computer searching skills. - Obtains the full text of articles and other evidence resources. - Understands the evidence written in English. - Uses available supports (healthcare librarians) to help find the evidence to answer the clinical question. - Employs bibliographic database tools (i.e., history, related articles, etc.). |  |
| **STEP 3. CRITICALLY APPRAISE THE EVIDENCE THAT HAS BEEN COLLECTED FOR ITS VALIDITY, RELIABILITY, AND APPLICABILITY, AND THEN SYNTHESIZE THAT EVIDENCE** | | | | |
| **Competencies** | **Learning outcomes related to EBP competency for nursing roles** | | | |
| - Critically appraises pre-appraised evidence (such as clinical practice guidelines, evidence-based policies and procedures, and evidence syntheses). - Critically appraises published research studies to determine their strength and applicability to clinical practice. - Evaluates and synthesizes a body of evidence gathered to determine its strength and applicability to clinical practice. - Leads a team to synthesize the evidence from primary research and pre-appraised evidence. | *Affective domain* | | - Encourages to evaluate clinical practice guidelines and other evidence for applicability and feasibility in practice. - Appreciates the practical utility of research findings. |  |
|  | *Cognitive domain* | | - Distinguishes the importance of the difference between evidence-based documents and opinion-based documents. - Describes the different quality level of the designs of the investigation studies. - Explains the principal measures of association and the potential impact that allow evaluating the magnitude of the analyzed effect in investigation studies. - ﻿Explains the purpose and processes of a qualitative study. - Identifies key criteria in any evidence reports using critical appraisal checklists. - Identifies the strengths and limitations of various types of research studies (quantitative and qualitative). - Identifies the biases in the principal investigation designs and the impact of these on the results. - Lists advantages of pre-appraisal documents as a strong evidential foundation for clinical decision-making. - Recognizes how qualitative research can inform the decision-making process. |  |
|  | *Skills domain* | | - Participates in institutional initiatives for evaluation and synthesis of a body of evidence gathered to determine its strength and applicability to clinical practice. - Assesses reliability, validity, and limitations of the research evidence. - Distinguishes the difference between clinical importance and statistical significance. - Creates or participates on teams oriented to synthesize evidence. - Grades the research studies results in the following level of evidence (quality) and grades (strength) of recommendation. - Interprets different types of measures of association and effect, including key graphical presentations and confidence intervals. - Uses relevant appraisal tools to evaluate the evidence. - Interprets the grading of the certainty in evidence and the strength of recommendations. |  |
| **STEP 4. INTEGRATE THE EVIDENCE WITH ONE**’**S CLINICAL EXPERTISE AND THE PATIENT**’**S PREFERENCES AND VALUES TO IMPLEMENT A CLINICAL DECISION** | | | | |
| **Competencies** | **Learning outcomes related to EBP competency for nursing roles** | | | |
| - Collects practice data (e.g., individual patient data, quality improvement data) systematically as internal evidence for clinical decision-making in the care of individuals, groups, and populations. - Integrates evidence gathered from external and internal sources in order to plan nursing care. - Implements practice changes based on evidence and clinical expertise and patient preferences to improve care processes and patient outcomes. - Leads transdisciplinary teams in applying synthesized evidence and internal evidence to initiate clinical decisions and practice changes to improve the health of individuals, groups, and populations. | *Affective domain* | | - Chooses evidence-based approaches over routine as a base for own clinical decision-making. - Considers the patients’ preferences and values when designing interventions or protocol changes. - Ensures that the delivery of care on the unit(s) or clinic(s) aligns with evidence-based practice recommendations. - Promotes practice changes based on evidence, clinical expertise, and patient preferences to improve care processes and patient outcomes. - Purposes modifications at the workplace/organization to implement. |  |
|  | *Cognitive domain* | | - Describes potential barriers and supports to knowledge translation and strategies to overcome these. - Identifies the components of the change process using a planned change model. |  |
|  | *Skills domain* | | - Interviews individuals and families to identify patient health status. - Adapts synthesized knowledge and recommendations from clinical practice guidelines to accommodate local clients, populations, and settings. - Creates or participates in an implementation plan for incorporating the consideration of best evidence into clinical practice. - Engages patients in the decision-making process, using shared decision-making, including explaining the evidence and integrating their preferences. - Creates strategies for supporting colleagues to implement practice changes. - Uses a comprehensive set of relevant variables within and across the system to measure the quality of care. - Uses organizational information (policies/guidelines, etc.) to change practice. - Changes clinical practice using guidelines/evidence-based protocols. - Changes the clinical practice based on patient assessment data. - Uses organizational quality indicators to identify health needs in patients. - Delivers care using evidence-based CPGs and another types of evidence. - Explains evidence and discusses options with the patient in lay language. - Updates nursing guidelines/standards/rules. - Leads or participates with the team to develop evidence-based practice recommendations for unit(s), clinic(s), and/or organization. - Manages or participates in an interdisciplinary team for implementing practice changes. |  |
| **STEP 5. EVALUATE OUTCOMES OF THE PRACTICE DECISION OR CHANGE BASED ON EVIDENCE** | | | | |
| **Competencies** | **Learning outcomes related to EBP competency for nursing roles** | | | |
| - Interprets obtained outcomes after the evaluation of an evidence-based change practice. - Measures processes and outcomes of evidence-based clinical decisions. - Generates internal evidence (evidence generated internally within a clinical setting, such as patient assessment data, outcomes management, and quality improvement data) through outcomes management and EBP implementation projects to integrate best practices. - Evaluates outcomes of evidence-based decisions and practice changes for individuals, groups, and populations to determine best practices. | *Affective domain* | | - Recognizes the need to evaluate the impact on outcomes. - Recognizes the importance of facilitating the evaluation (register) for the EBP. |  |
|  | *Cognitive domain* | | - Describes an evaluation plan to analyze the changes produced. - Identifies data and indicators to evaluate services for individuals, families, and groups. - Identifies a strategy for direct measures of care outcomes (e.g., derived from clinical documentation, case review, patient's feedback). - Relates cost outcomes and patient benefits. - Describes specifically safety and quality outcomes of nursing care. |  |
|  | *Skills domain* | | - Interprets analysis of indicators/outcomes in terms of quality of care. - Assesses the effectiveness of the interventions to determine improvement in patients or practice. - Changes practice based on patient outcome data. - Collects practice data (e.g., individual patient data, quality improvement data) systematically for clinical decision-making in the care of individuals, groups, and populations. - Manages the interdisciplinary team for outcomes evaluation. - Evaluates the application of interventions and identifies areas for improvement. - Implements processes to monitor and evaluate the impact of practice change (individual, service, and organization). - Participates in evidence-based quality improvement processes to evaluate outcomes of practice changes. - Participates in the review of practice outcomes, standards, and guidelines; review of policies, procedures, and guidelines based on evidence. - Uses audit and feedback of data as an implementation strategy to promote the use of the evidence-based practice in the unit(s) or clinic(s). |  |
| **STEP 6. DISSEMINATE THE OUTCOMES OF THE EBP DECISION OR CHANGE** | | | | |
| **Competencies** | **Learning outcomes related to EBP competency for nursing roles** | | | |
| - Disseminates best practices supported by evidence to improve quality of care and patient outcomes. - Formulates evidence-based policies and procedures. - Leads or participates in the generation of external evidence with other healthcare professionals. - Communicates best evidence to individuals, groups, colleagues, and policymakers**.** | *Affective domain* | | - Performs activities to disseminate EBP. - Believes in the importance of sharing results of practice changes with colleagues, patients, and stakeholders. - Encourages experience sharing to emphasize the need for change and positive outcomes of change. |  |
|  | *Cognitive domain* | | - Defines a variety of methods to disseminate results of practice changes tailored to communities/populations. - Describes ethical, legal, and policy guidelines in the dissemination of data and information. - Identifies the principal sections of scientific communication (oral and poster presentations, papers, etc.). - Lists peer-reviewed journals and national-level meetings for dissemination of evidence-based practice outcomes. |  |
|  | *Skills domain* | | - Creates strategies for dissemination of evidence-based practice into the healthcare environment. - Prepares (or participates in) academic writings for results dissemination. - Synthesizes (or participates in) the results of practice change in an understandable way. - Shares (or participates in) structural, process, and patient outcomes from an EBP implementation project. - Gives feedback regarding patient outcomes and achievements to colleagues in a constructive way. - Utilizes the information and communications technology in sharing results of practice changes. - Adapts (or participates in) the communication of obtained outcomes to the different audiences (patients, colleagues, policymakers) and/or media and audiences. - Demonstrates public speaking and active listening skills. - Leads or participates in interdisciplinary teams, including patients and professional associations, to the dissemination of outcomes. - Discusses implications of research with colleagues. |  |
